# Questioning the Claimed Superiority of Malaria Parasite Ex Vivo Viability Reduction Over Observed Parasite Clearance Rate?

**DOI:** 10.1093/infdis/jiaa790

**Published:** 2020-12-28

**Authors:** Nicholas J White, James A Watson

**Affiliations:** Mahidol Oxford Research Unit, Faculty of Tropical Medicine, Mahidol University, Bangkok, Thailand

To the Editor—In a study of 10 *Plasmodium falciparum*–infected volunteers with submicroscopic parasitemias given a single 200-mg dose of artesunate, Rebelo et al [1] reported a substantial difference in the ex vivo growth of sequentially sampled circulating ring-stage [2] parasites comparing infections with artemisinin-sensitive (Pfkelch wild-type) and artemisinin-resistant (Pfkelch R539T) parasites. In the 5 artemisinin-sensitive infections, they derived an estimated ex vivo mean parasite “viability” reduction half-life of 0.75 hour, considerably shorter than the corresponding 3.2-hour in vivo mean parasite clearance half-life estimate. In contrast, in the 5 artemisinin-resistant infections, the mean estimated viability reduction half-life was 8.7 hours, compared with 6.5 hours for the in vivo parasite clearance half-life.

This observation is consistent with numerous laboratory studies showing that artemisinin resistance in *P. falciparum* is associated with loss of ring-stage susceptibility [3–6]. Indeed, this clinical study can be considered as a rather laborious in vivo ring-stage survival assay [4]. The “viability” effect measure is derived from the subsequent ex vivo growth of malaria parasites following different drug exposures. The reduction in viability reflects the damage done by the drug exposure in vivo, and any parasite sequestered antimalarial drug in the ex vivo culture, and the continued effects of that damage. This was compared with the serial parasite densities at the time of blood sampling, which are used to provide a parasite clearance rate [7].

The serial quantitative polymerase chain reaction derived parasitemia profiles shown by Rebelo et al [1], fig 3 strongly suggest continued input into the circulation from ongoing schizogony [2, 8]. This explains why parasite densities in blood do not fall for approximately 8 hours. The most commonly used parasite clearance rate estimator explicitly accounts for this lag-phase [7]. In contrast, the viability estimates use blood samples containing circulating parasites and high artesunate concentrations, and much of the effect is observed by the first sampling time point (2 hours). Taking a blood sample and diluting out the antimalarial drug does not instantly stop it working. Parasites take time to die, so it is not surprising that the ex vivo assessment over days suggests greater “killing” than the densities of parasites in the blood at the time of sampling would suggest, but to conclude that “parasite resistance to artemisinins may have a more profound effect on in vivo drug efficacy than previously appreciated” is not warranted. If this means that parasite killing by artemisinins has been underestimated, then it is not compatible with clinical trial observations of the relationship between dosing, duration of treatment, and outcome [9].

The title of the article, “Parasite viability as a superior measure of antimalarial drug activity in humans” [1], suggests a significant advance, but it is not clear why or how it would be used to assess antimalarial drugs. It is stated that “the use of parasite clearance to measure drug activity and to inform decisions about drug development should be reconsidered in view of these new insights.” It is unclear what these insights are and whether these difficult and laborious serial in vivo studies would offer any advantage over the currently used, simple ring-stage in vitro tests [4, 6], which identify the loss of ring-stage activity in artemisinin-resistant parasites very well.

The meaning and predictive value of the estimated half-life from the viability studies are also unclear. The observed log-linear decline in parasite densities in blood after artemisinin treatment provides a clearance half-life of about 3.5 hours, which, if continued, would result in an approximately 16 000-fold decrease per life-cycle. This predicts that ≥5 days of artemisinin monotherapy (regardless of dosing frequency) are needed to clear an infection with a biomass of 10^12^ parasites. This matches clinical observations [9]. But what is the meaning or utility of the half-life estimated from the viability study? Interpreted literally, a continued half-life of 0.75 hours would kill all the infecting malaria parasites within a day, which clearly does not match clinical observations.

As for dose finding, the results presented in [1] fig 3 suggest that the fits to the serial viability log-linear declines are poor and, thus, the derived viability half-lives are imprecise in comparison with the parasite clearance profiles. Indeed, it is unclear whether declines are exponential and, therefore, whether the model is appropriate. This does not give confidence that a concentration-effect (dose-response) estimate derived from these viability data will be more informative than one derived from parasite clearance profiles. Serial circulating malaria parasite viability estimations are certainly unsuited for field assessments and, importantly, they are not relevant for the majority of current antimalarial drugs, which have little or no effects on ring-stage parasites.

## Notes

***Financial support.*** This work was supported by the Wellcome Trust.

***Potential conflicts of interest.*** Both authors: No reported conflicts. Both authors have submitted the ICMJE Form for Disclosure of Potential Conflicts of Interest. Conflicts that the editors consider relevant to the content of the manuscript have been disclosed.
